# Clinical Significance of the Radiological Relationship between the Tumor and the main blood vessels in Enneking IIB Osteosarcoma of the extremities

**DOI:** 10.7150/jca.42341

**Published:** 2020-03-05

**Authors:** Qinglin Jin, Xianbiao Xie, Hao Yao, Lili Wen, Hongbo Li, Dongming Lv, Ziliang Zeng, Yongqian Wang, Changye Zou, Junqiang Yin, Gang Huang, Bo Wang, Jingnan Shen

**Affiliations:** 1 Department of Musculoskeletal Oncology Center, The First Affiliated Hospital of Sun Yat-sen University, 58 Zhongshan 2 nd Rd, Guangzhou 510080, China.; 2 Guangdong Provincial Key Laboratory of Orthopedics and Traumatology, Guangzhou 510080, China.; 3 Department of Anesthesiology, State Key Laboratory of Oncology in South China, Sun Yat-sen University Cancer Center, 651 Dongfengdong Rd, Guangzhou 510060, China.

**Keywords:** Osteosarcoma, Enneking ⅡB, Reactive Area, Risk Factor, Radiological Vascular Involvement

## Abstract

**Aim**: Osteosarcoma is one of the most prevalent primary bone malignancies in children and adolescents. Magnetic resonance imaging (MRI) has been considered a very critical tool to provide anatomical information of tumor and surrounding main blood vessels. To evaluate the prognostic significance of the radiological vascular involvement according to the pre-treatment MRI in patients with Enneking IIB osteosarcoma.

**Methods**: In this retrospective study, we included 482 patients younger than 50 years old with Enneking IIB primary osteosarcoma of the extremities with complete clinical records from 2005 to 2015.Univariate and multivariable analyses were conducted to identify the risk factors for OS (Overall survival) and EFS (Event-free survival). The correlations between the risk factors was performed using Spearman analysis. The Kaplan-Meier method was used to calculate survival curves. Based on the radiological relationship between the tumor lesion and the surrounding reactive area with the main blood vessels as shown on pretreatment MRI findings.

**Results**: Radiological vascular involvement assessed via pretreatment MRI is an important risk factor for Enneking IIB primary patients with osteosarcoma (HR_OS_=2.32/HR_EFS_=1.81 P<0.01) according to the univariate and multivariable analyses. Enneking IIB patients with osteosarcoma were assigned to three subtypes based on the radiological relationship between the main blood vessels and the lesion or reactive area. The 5-year cumulative OS of patients classified by the three types were 81.6% (type I), 67.1% (type II) and 44.8% (type III)(P<0.01). The 5-year cumulative EFS of the three types were 60.2% (type I), 46.7% (type II) and 30.2% (type III)(P<0.05). The total 5-year cumulative OS and EFS for all patients were 68.3% and 48.3%, respectively.

**Conclusion**: Vascular involvement according to radiological findings from pretreatment MRI is an independent risk factor for cumulative OS and EFS in patients with Enneking IIB primary osteosarcoma of the extremities. The new subtyping based on the relationship between the tumors and surrounding reactive area with the main blood vessels based on pretreatment MRI can predict the prognosis of patients with osteosarcoma and provide certain directive information for selecting the appropriate surgical procedure for individual patients.

## Introduction

Osteosarcoma is one of the most prevalent primary bone malignancies in children and adolescents 3. Collectively, osteosarcoma usually originates from the metaphysis of long bones, and the distal femur and proximal tibia are the most common sites of osteosarcoma of the extremities, which comprises over 60% of the reported osteosarcoma diagnoses. With the help of long-standing neoadjuvant and adjuvant chemotherapy, the 5-year cumulative survival rate of osteosarcoma has been elevated to more than 60% since the 1970s 22,24,26.

Enneking et al proposed a classical staging system for malignant bone tumors. This staging system has been widely used by musculoskeletal oncologists to assess the prognosis of patients with osteosarcoma, which also has surgical implications 9. The vast majority of patients with osteosarcoma are assigned to Enneking stage IIB. However, the prognosis of patients in this group is not uniform. It is not enough to evaluate the prognosis of patients with osteosarcoma or make surgical decisions using the Enneking staging system alone in this group of patients. Increasing evidence indicates that numerous risk factors have been associated with the prognosis of Enneking IIB osteosarcoma. 1-2,4,16-17,19,21. These risk factors have been individually used in or combined into models to predict the prognosis of Enneking IIB patients with osteosarcoma 14,18,28,30,32. However, most of those prediction models are utilized after the surgical procedure. The optimal pretreatment prediction model still challenges musculoskeletal oncologists.

Magnetic resonance imaging (MRI) has been considered a very critical imaging tool to provide anatomical information on patients with osteosarcoma upon initial diagnosis. MRI can depict the relationship between the tumor and adjacent neurovascular bundles and the surrounding reactive area 10,34. Radiological vascular involvement detected by MRI is a risk factor for the prognosis of soft tissue sarcoma 25. Pretreatment assessments based on MRI data are useful for surgical decisions in soft tissue sarcoma (STS) 5,12,27.

In this study, we first investigated whether radiological involvement of the main blood vessels assessed by MRI is an important prognostic risk factor of patients with Enneking IIB osteosarcoma. Then, we proposed subtyping of Enneking IIB osteosarcoma of extremities based on the relationship between the tumor lesion and surrounding reactive area with the main blood vessels in the pre-treatment MRI, assess its prognostic significance in osteosarcoma patients and potential guidance in selecting surgical procedure individually.

## Methods

### Study population

In this retrospective study, records of 786 patients who were younger than 50 years old at the time of diagnosis and who were newly diagnosed with osteosarcoma and received standard treatment according to National Comprehensive Cancer Network (NCCN) guidelines from January 2005 to December 2015 at our institution are reviewed.

The inclusion criteria were as follows: (1) patients with histologically confirmed (by needle biopsy) primary high-grade osteosarcoma; (2) patients with tumors located in the extremities; (3) patients who did not receive any antitumor therapy before admission to our center; (4) patients with complete clinical, imaging, pathological and follow-up data; (5) patients who received standard treatment 9.

The exclusion criteria were as follows: (1) patients with evidence of underlying diseases such as hypertension, diabetes, cardiac or pulmonary diseases; (2) patients suffering from another type of tumor or secondary osteosarcoma; (3) patients who received specific treatment in other case-control studies; (4) patients with Enneking stage ⅡA or Ⅲ at the time of diagnosis; (5) patients with a Karnofsky performance score<70; (6) patients' age above 50 years old 20.

After applying the inclusion and exclusion criteria, we finally enrolled 314 (65.1%) patients with distal femoral osteosarcoma, 117 (24.3%) patients with proximal tibial osteosarcoma, 28 (5.8%) patients with proximal humeral osteosarcoma and 23 (4.8%) patients with osteosarcoma at other sites (Table 1).

**Figure 1:**
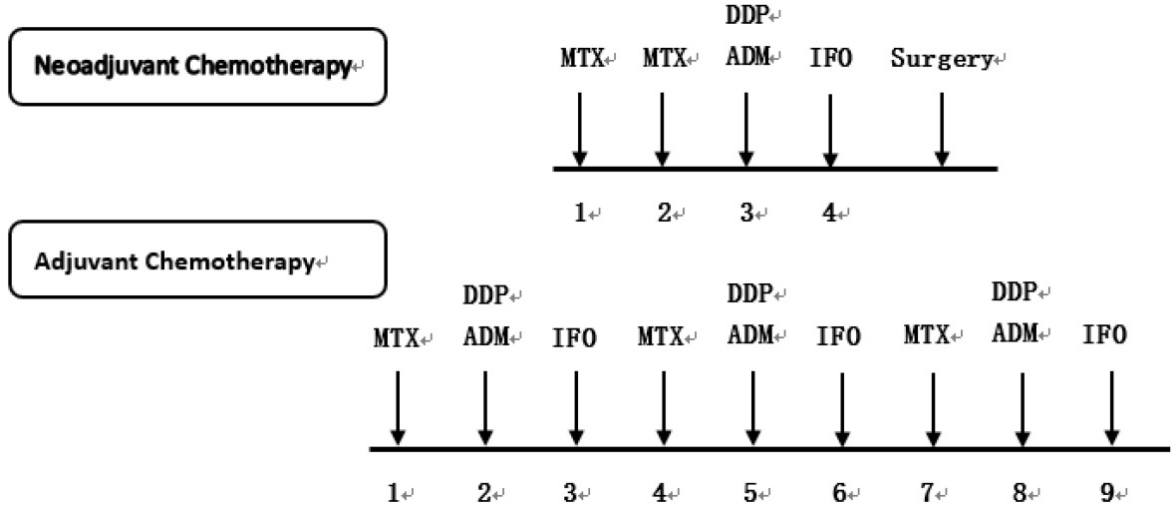
** Chemotherapeutic agents and treatment protocol used in 482 patients with osteosarcoma.** MTX: Methotrexate, DDP: Cisplatin, ADM: Doxorubicin, IFO: Ifosfamide.

Diagnoses of osteosarcoma and pulmonary metastasis are based on multidisciplinary factors. X-ray imaging and MRI were performed for local lesions to evaluate tumor size and aggressiveness. A pretreatment needle biopsy of the primary lesion was performed for each patient to confirm the pathological stage of osteosarcoma. Computed tomography (CT) scan of the lung and/or a Positron Emission Tomography-Computed Tomography (PET-CT) scan were also performed to evaluate pulmonary or distant metastasis for osteosarcoma to confirm the Enneking stage. Standard treatment of osteosarcoma involved neoadjuvant chemotherapy, surgery and adjuvant chemotherapy. Four commonly used drugs for neoadjuvant and adjuvant chemotherapy include high-dose methotrexate, cisplatin, doxorubicin, and ifosfamide, which were administered at the interval presented in Figure 1 33.

### Follow-up

Patients were followed up every 3 months for the first and second years, every 4 months for the third year, every 6 months for years 4 and 5, and yearly thereafter. For every follow-up, patient history was taken, and physical examination, X-ray imaging of the lesion site and CT scan of the lungs were performed; PET-CT was performed if potential metastasis or recurrence was noticed.

### MR imaging

All patients underwent pretreatment MRI on a 3.0-T MRI system (MR scanners: (1) Siemens Magnetom Trio, Erlangen, Germany; (2) Siemens Magnetom Verio, Erlangen, Germany) with a dedicated eight-channel surface coil or a body coil, depending on the anatomic disease site. MRI acquisition in all cases included sagittal, coronal and transversal T1-weighted (TR 540-750 ms/TE 11-18 ms) and fast spin echo T2-weighted (TR 3500-6460 ms/TE 81-100 ms, echo train length [ETL] 8) with fat suppressed images.

### Methods of evaluation

Blood samples were obtained just prior to the initial neoadjuvant chemotherapeutic treatment to measure the alkaline phosphatase (ALP) and lactic dehydrogenase (LDH) levels. Patients were categorized into two distinct age groups (0-14 years; >14 years) 2. According to the American Joint Committee on Cancer (AJCC), we categorized the maximum tumor diameter into two distinct groups (0-80 mm; >80 mm) 17.

Distinguishing tumor from the peritumoral reactive area was determined according to Lawrence M's criteria 31. The maximum diameter of the tumor was defined as the longest diameter of the largest cross-sectional area. The main blood vessels included the main branches of the axillary, brachial, radial, ulnar, femoral, popliteal and posterior tibial artery and vein.

**Figure 2:**
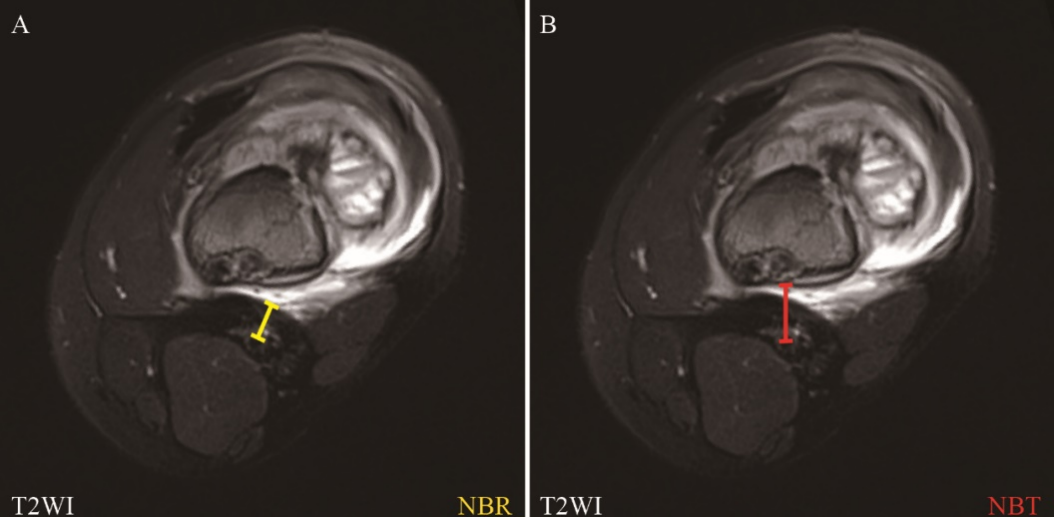
** Assessment of the nearest of blood vessels to reactive zone (NBR) and the nearest of blood vessels to the tumor (NBT) in MRI.** NBR: the nearest of blood vessels to reactive zone; NBT: the nearest of blood vessels to the tumor.

Here, we defined radiological vascular involvement as that when MRI does not show a rim of normal tissue in the tumor/reactive area-to-vessel interface, even if any part of the main blood vessels pass through the tumor lesion or reactive area on the MRI findings 27. The distance between the tumor lesion, reactive area and main blood vessels on the pretreatment MRI were measured as previously described 33. We defined NBR: the shortest distance from the center of the main blood vessels to the margin of the reactive area; NBT: the shortest distance from the center of main blood vessels to the margin of tumor lesion (Figure 2). NBR or NBT was noted as 0.00 cm when the main blood vessels were radiologically involved by reactive area or tumor lesion, respectively. The measurement and further subtyping of the radiological relationship between the main blood vessels and tumor lesions or reactive areas was performed independently by two medical imaging specialists, and discussions were carried out when disagreements occurred until achieving a consensus.

### Primary outcomes

Overall survival (OS) is defined as the time from diagnosis until death from various causes or until the last follow-up visit.

Event-free survival (EFS) is defined as the time from diagnosis until any event related to the tumor, such as local recurrence, distant metastasis, second primary malignancy, death, etc.

### Statistical analysis

SPSS (IBM corp. 22, New York, USA) statistical software was used for statistical analysis. Categorical variables are expressed as numbers and percentages, and the χ^2^ test was used to compare differences between groups. Continuous variables are presented as the mean (range), and means were compared via ANOVA (Analysis of variance). The correlations between the risk factors were performed using Spearman analysis. Univariate and multivariable analyses were performed to identify the probable prognostic factors. Variables that were found to be significant in the univariate analysis were included in the multivariable analysis with the Cox proportional hazard model. The Kaplan-Meier method was used to calculate survival curves to estimate OS and EFS; differences between the curves were evaluated with the log-rank test. A P-value<0.05 was considered statistically significant. Intra-observer and interobserver agreement were assessed by using the Cohen's kappa statistics. The strength of the kappa agreement was defined as follows: 0.00-0.20 indicated sight agreement; 0.21-0.40 fair agreement; 0.41-0.60, moderate agreement; 0.61-0.80, substantial agreement; and 0.81-1.00, almost perfect agreement.

## Results

### Patients' characteristics

A total of 482 patients were finally included in our study. The mean age at the time of diagnosis was 19 (range: 7-47) years old, among whom 298 were males (61.8%), and the median follow-up time was 60.0 (range: 28-161) months. Primary site of lesion included distal femur (n = 314 [65.1%]), proximal tibia (n = 117 [24.3%]) and proximal humerus (n = 28 [5.8%]). The mean maximum lesion diameter was 78.3 (range: 38-105) mm. The mean ALP and LDH levels were 234 (range: 49-1546) U/L and 256 (range: 77-1122) U/L, respectively. Furthermore, radiological vascular involvement (RVI) of the main blood vessels by tumor lesions was detected in 32 (6.6%) patients, while the main blood vessels of 450 (93.4%) patients were free from tumor lesions. Eighty-eight (19.5%) patients underwent amputation, and 394 (81.7%) patients in this study received limb-salvaging surgery (Table 1).

### Radiological vascular involvement is an independent risk factor for prognosis in Enneking IIB patients with osteosarcoma

**Figure 3:**
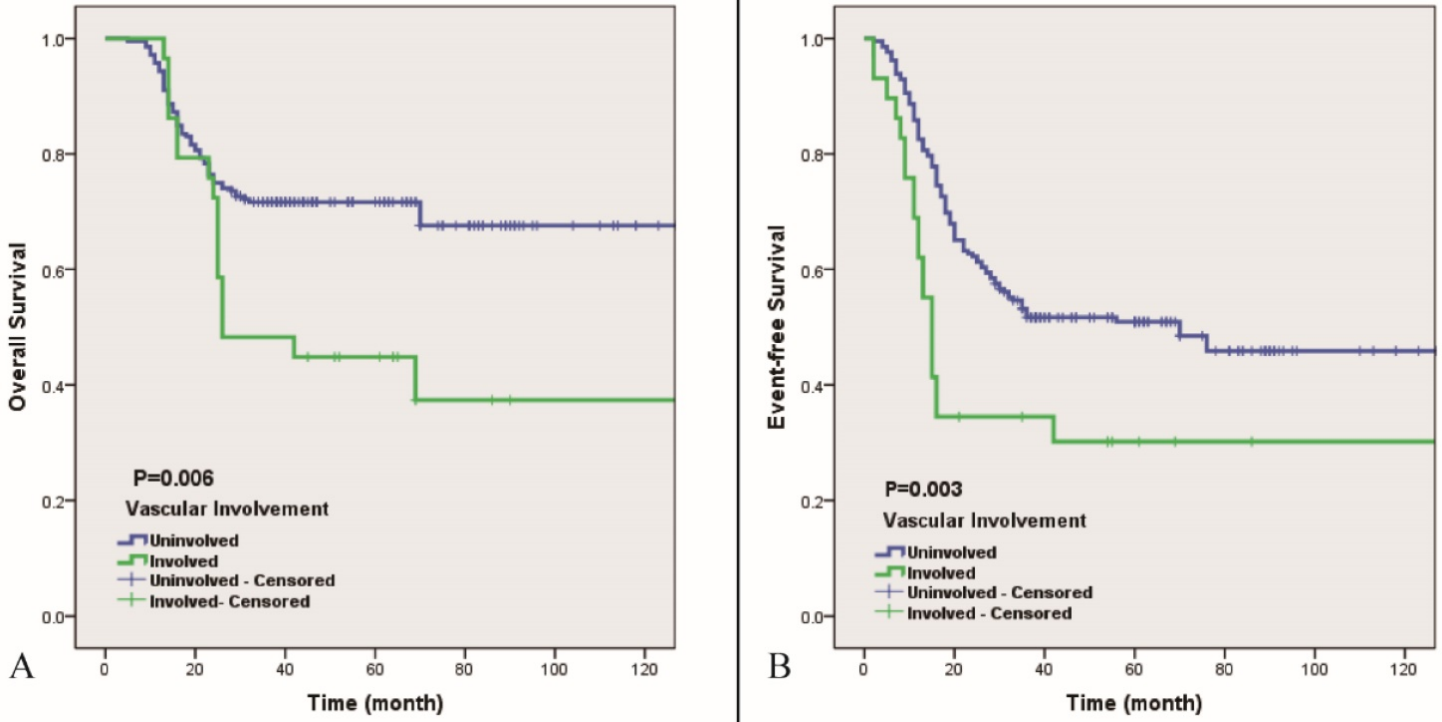
** 5-year OS (A) and EFS (B) of vascular involvement of Enneking IIB osteosarcoma of extremities.** Blue line indicates uninvolved, green line indicates involved. Involved means MR imaging does not show a rim of normal tissue in the tumor-to-vessel interface (P<0.01).

The results of the univariate analysis of the patients are listed in Table 2. According to the univariate analysis, several potential risk factors are closely related to the 5-year cumulative OS and EFS such as the maximum diameter of the tumor lesion, pretreatment ALP levels and radiological vascular involvement (P<0.05) (Table 2). Then, we further analyzed those independent risk factors via multivariable analysis using a Cox proportional hazard model. As Table 3 shows, interestingly, the maximum diameter of the tumor lesion is most strongly related to the 5-year cumulative OS (Hazard ratio (HR)=2.32, P<0.01), while radiological vascular involvement is the strongest independent risk factor for the 5-year cumulative EFS (HR=1.81, P<0.01). Figure 3 shows the Kaplan-Meier curve indicating that radiological vascular involvement by tumor lesions is highly related to 5-year cumulative OS and EFS (P<0.01). The 5-year OS and EFS of the group without radiological vascular involvement (the uninvolved group) were 71.7% and 50.9%, respectively, which were significantly higher than those of the group with radiological vascular involvement (the involved group) (44.8% and 30.2%, respectively).

### Subtyping of Enneking IIB osteosarcoma based on the radiological relationship between the tumor and peritumoral reactive area with the main blood vessel

**Figure 4:**
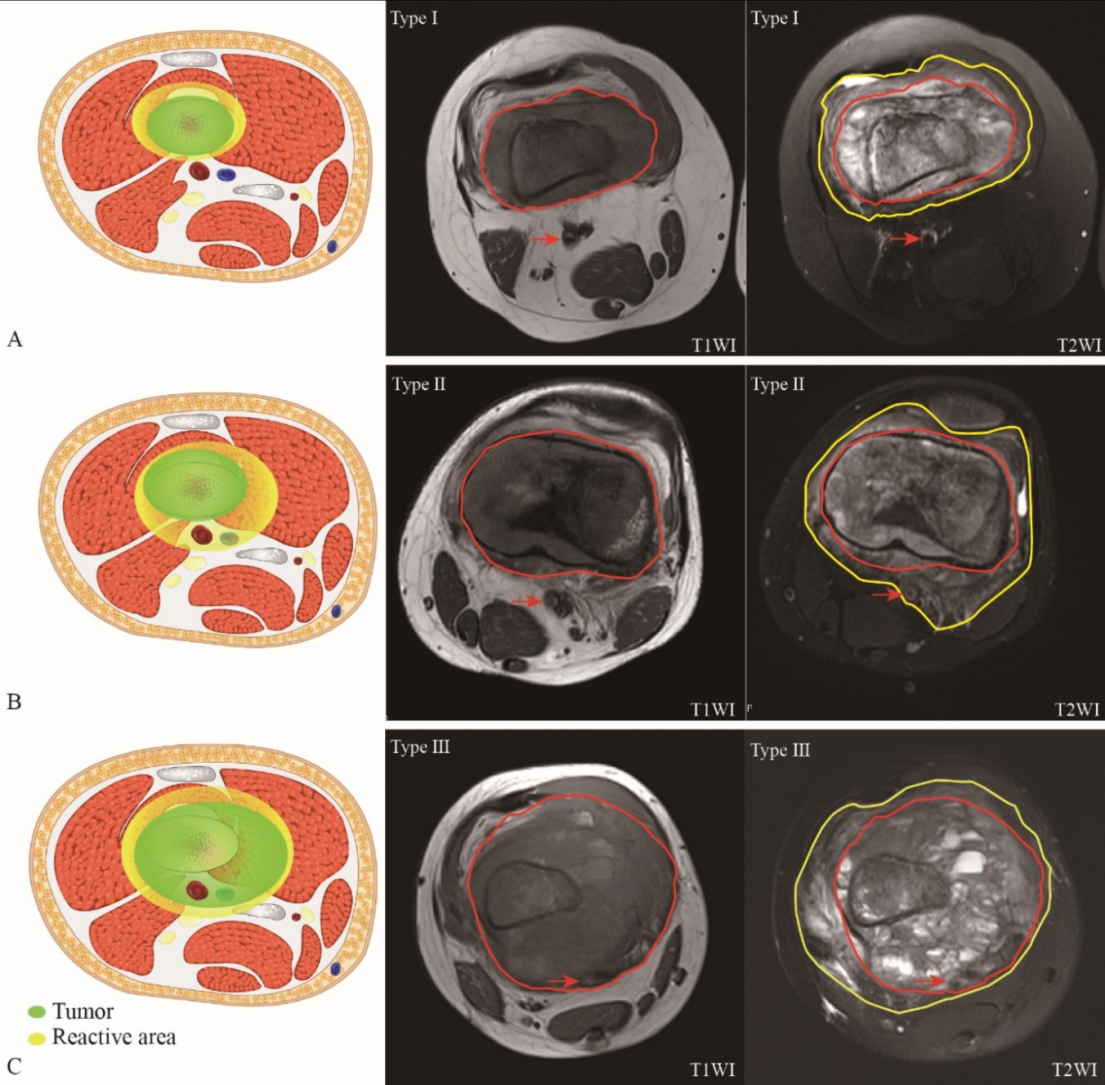
** MRI of three subtypes of Enneking IIB osteosarcoma with schematic diagram (3A, 3B, 3C).** In the schematic diagram (left), green area indicates tumor lesion while yellow are indicates reactive area. In the MRI, area surrounded by red indicates tumor lesion in T1WI (middle) and T2WI (right); area surrounded by yellow line indicates peritumoral reactive area in T2WI (right); red arrow in MRI indicates main blood vessels.

Vascular involvement is a strong independent risk factor for both 5-year cumulative OS and EFS. Furthermore, we subdivided patients with Enneking ⅡB osteosarcoma into three different subtypes with the help of MRI data: type I, all main blood vessels are free from the reactive area (NBR>0.00 cm, NBT>0.00 cm); type II, at least one main blood vessel is radiologically involved with the reactive area, while none of main blood vessels are radiologically involved with the tumor lesion (NBR=0.00 cm, NBT>0.00 cm); type III, at least one main blood vessel is radiologically involved with the tumor lesion partly or wholly (NBR=0.00cm, NBT=0.00 cm). Figure 4 shows the different subtypes of Enneking IIB osteosarcoma with a schematic diagram. Obviously, the green and yellow arrows in the MR image indicate tumor lesions and peritumoral reactive areas, respectively. In the schematic diagram, the green area indicates the tumor lesion, and the yellow area indicates the peritumoral reactive area, which corresponds with the MR image.

Most patients were diagnosed with type II (404, 83.9%), followed by type I (46, 9.5%) and type III (32, 6.6%) (Table 4). The mean ages at the time of diagnosis of the three subtypes were 23 (range: 11-47), 19 (range: 7-47) and 16 (range: 8-29) years old, respectively (P<0.05). Most patients had tumors located in the distal femur in type I (65.2%) and type II (68.1%), while most type III (37.5%) patients with osteosarcoma had tumors localized to other sites. The mean maximum diameters of the lesions were 63.2 (range: 39-104) mm, 79.1 (range: 38-105) mm and 84.0 (range: 44-97) mm, respectively (P<0.05). The mean NBR were 0.38 (0.31-0.58) cm (type I), 0.00 cm (type II) and 0.00 cm (type III) while the mean NBT were 1.07 (0.69-1.46) cm (type I), 0.35 (0.28-0.44) cm (type II) and 0.00 cm (type III), respectively (P<0.05).The mean ALP and LDH levels were also significantly different in the three types of patients. The mean ALP and LDH levels were 179 (range: 52-397) U/L and 225 (range: 86-681) U/L in type I, respectively, which are the lowest among the three subgroups. Furthermore, most patients with type III underwent amputation (n = 28, [87.5%]), while only 2 patients of type I underwent amputation surgery (4.3%).

### Clinical significance of pretreatment MRI-based radiological subtyping in in Enneking IIB patients with osteosarcoma

**Figure 5:**
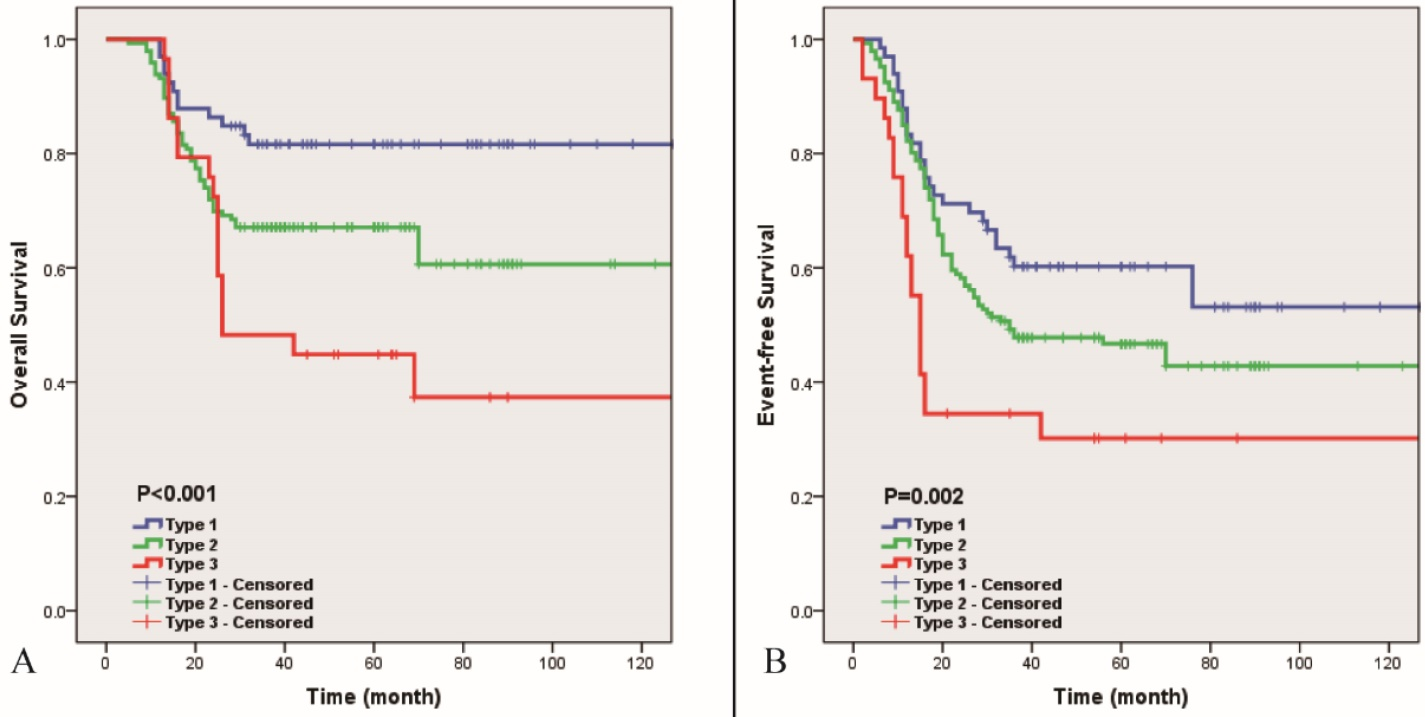
** 5-year OS (4A) and EFS (4B) of 3 subtypes of Enneking IIB osteosarcoma.** Blue line indicates Type I, green line indicates Type II and red line indicates Type III (P<0.01).

To study whether the subtypes of Enneking IIB osteosarcoma defined by the radiological relationship between blood vessels and the lesion or reactive area shown on MRI have differences in survival, we analyzed the 5-year cumulative OS of the three subtypes: 81.6% (type Ⅰ), 67.1% (type II), and 44.8% (type III), while the total 5-year cumulative OS of Enneking IIB osteosarcoma was 68.3% (P<0.01). Additionally, we analyzed the 5-year cumulative EFS of the three subtypes: 60.2% (type I), 46.7% (type II), and 30.2% (type III). The total EFS of Enneking IIB osteosarcoma was 48.3% in this study (P <0.01) (Figure 5).

**Figure 6:**
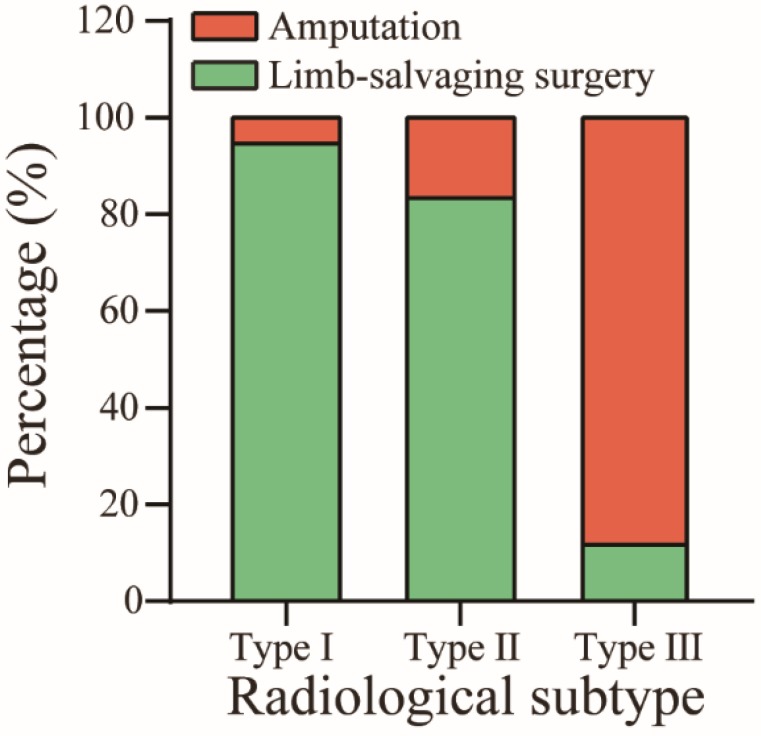
Percentage of limb-salvaging and amputation surgery in three radiological classification groups.

In this study, the overall amputation rate was 19.5% (Table 1). The rates of amputation in the subtypes were 4.3% (type I), 15.8% (type Ⅱ), and 87.5% (type III) (P < 0.01) (Figure 6). One of the type I patients received amputation surgery due to his poor economic condition in which he could not afford high-priced endoprostheses. For the other young type I patient who decided to receive amputation, the patient and family urged amputation because they were worried about the recurrence of the tumor after limb-salvaging surgery. Most type II patients underwent amputation because there was not enough soft tissue to reconstruct after tumor resection. For four type III patients who received limb-salvaging surgery, they all strongly required salvaging the limb. Therefore, we tried to resect the tumor completely and separate the partly adhered main blood vessel while excising the vascular sheath in two patients. For the other two whose main blood vessels were completely encompassed by the tumor lesion, we resected the tumor and the involved main blood vessel and utilized a vascular graft to repair the limbs.

### Intraobserver and interobserver agreement

With regard to the intraobserver agreement, substantial agreements were observed for subdivision of radiological types (radiologist 1: kappa=0.683, 95% CI: 0.462-0.904; radiologist 2: kappa= 0.692, 95% CI: 0.673-0.901). For interobserver agreement between the two radiologists, almost perfect agreement was observed (kappa = 0.595, 95% CI: 0.521-0.668) (Table 5).

## Discussion

In this study, we investigated the relationships between different types of radiological vascular involvement and survival outcomes for Enneking ⅡB osteosarcoma of the extremities. To date, the Enneking staging system has been widely accepted and applied by musculoskeletal oncologists throughout the world as a standard classification. However, the majority of patients diagnosed with Enneking ⅡB osteosarcoma have variable prognoses. Bacci, G et al 1 demonstrated that pretreatment LDH levels should be considered when deciding on a risk-adapted treatment for OS patients. In addition, Bacci,G et al 2 emphasized that age younger than 14 years, high levels of serum ALP, and inadequate surgical margins should be considered when deciding risk-adapted treatment options for patients with osteosarcoma. Kim, M.S.et al 17 suggested that an 80 mm maximal cutoff is strongly useful for subdividing patients with osteosarcoma. According to the Birmingham classification of osteosarcoma 14, a combination of the recording of surgical margins in millimeters and the response to neoadjuvant chemotherapy is likely to be more accurate and useful than the current MSTS system for Enneking ⅡB osteosarcoma. In addition to these factors, main blood vessel involvement was recently considered to be a critical prognostic risk factor in many types of malignant tumors. Kato, T. et al 15 emphasized that patients with blood vessel invasion had significantly worse recurrence-free survival and overall survival in primary invasive breast cancer with a hazard ratio of 2.1 (P<0.05). In high-grade soft tissue sarcoma, patients with histologically determined vascular involvement had a worse prognosis, and 70% of these patients experienced metastasis, with a 5-year metastasis-free survival of only 28% 25. Carneiro, A. et al 5 combined tumor size, vascular invasion, necrosis and growth pattern (SING) into a favorable model that could predict the prognosis of soft tissue sarcoma patients with a sensitivity of 74% and a specificity of 85%. Histological vascular invasion is defined as the presence of tumor cells within any space of the vascular wall or within the lumen of blood vessels 5, 11. On another words, oncologists can only perform these assessments postoperatively. Additionally, vascular involvement is also important for making surgical decisions, such as choosing between limb-salvaging surgery or amputation. Therefore, evaluating the condition of the main blood vessels before surgery is a critical procedure 23,29.

Magnetic resonance imaging (MRI) is an accurate and prevalent imaging modality for depicting tumor lesions, peritumoral reactive area and encroachment of tumors on adjacent neurovascular bundles 25. Holzapfel, K., et al 13 demonstrated that histological vascular invasion should be highly suspected when the lesion partially or completely covers the main blood vessels on MRI, and when combined with postoperative pathology, it was found that the sensitivity and specificity of MRI in judging whether the tumor was invaded by blood vessels were approximately 84.6% and 97.5%, respectively. The radiological vascular involvement demonstrated on pretreatment MRI in Enneking IIB primary patients with osteosarcoma has predictive ability for 5-year overall survival and event-free survival on both uni- and multivariable analyses. Based on our results, the prognosis of patients with osteosarcoma with tumor lesion involvement of the main vessels on MRI was significantly worse than that of patients without blood vessel involvement. The 5-year OS and EFS of the uninvolved group were 71.7% and 50.9%, whilst those of the involved group were 44.8% and 30.2%, respectively. Therefore, radiological vascular involvement is an independent risk factor of prognosis in Enneking IIB patients with osteosarcoma.

Tumor cells or satellite lesions may exist within the reactive area detected by MRI, which might involve the main blood vessels and worsen the prognosis of patients with malignancies 7. As reported by He, F., et al 12, the tumor and peritumoral reactive area measured by MRI are independent risk factors for predicting the prognosis of Enneking IIB patients with osteosarcoma who receive limb-salvaging surgery. White et al. reported that a single or cluster of tumor cells were found over 25% of specimens taken in reactive zone 31.Moreover, Cheon, H, et al 6 demonstrated that peritumoral edema detected by MRI was found in a significantly higher proportion of breast cancer patients with tumor recurrence (50%, P=0.022). In this study, we further subdivided patients into the uninvolved group, according to the relationship between the main blood vessel and peritumoral reactive area, and found that the prognosis of patients with main vessels passing through the reactive area was worse than that of patients without. Finally, patients in our study were assigned to three subtypes, and the 5-year cumulative OS rates of the three subtypes were 81.6% (type I), 67.1% (type II), and 44.8% (type III). The EFS rates of the three subtypes were 60.2% (type I), 46.7% (type II), and 30.2% (type III).

According to the pretreatment MRI-based radiological relationship between the main blood vessels and the lesion or reactive area, we classified Enneking IIB osteosarcoma into three subtypes: type Ⅰ, all main blood vessels are free from the reactive area; type II, at least one main blood vessel is radiologically involved with the reactive area, while none of the main blood vessels are radiologically involved with the lesion; type III, at least one main blood vessel has already been radiologically involved with the lesion partly or wholly. The prognosis of type I patients is the best, while that of type III patients is the worst. This radiological classification procedure via MRI data before treatment is a simple, rapid and noninvasive examination. In addition, surgeons can investigate the initial relationship between the tumor (or reactive area) and the main blood vessels before any changes in involvement are implemented by treatment or examination. Naturally, this new classification can probably provide some initial clues for making surgical decisions. There are several limitations in this study. First, all the patients included were from a single center, which means the findings still need to be validated prior to the application of this classification in other centers. Second, it's a retrospective study. Third, we did not evaluate functional assessment in this study.

## Conclusion

In conclusion, our study results suggest that the presence of radiological vascular involvement of main blood vessels identified by preoperative MRI is an independent factor associated with the prognosis of Enneking IIB patients with osteosarcoma. The assessment of radiological classification before treatment can provide prognostication for patients with Enneking IIB osteosarcoma, as well as a helpful classification for making surgical decisions for Enneking IIB patients with osteosarcoma.

## Figures and Tables

**Table 1:** Baseline demographic characteristics of the study group

Characteristics	
Mean age (Years, Range)	19 (7-47)
**Gender (N, %)**	
Male	298 (61.8)
Female	184 (38.2)
**Site of tumor (N, %)**	
Distal Femur	314 (65.1)
Proximal Tibia	117 (24.3)
Proximal Humerus	28 (5.8)
Others	23 (4.8)
Maximum diameter of tumor (mm, Range)	78.3 (38-105)
Mean ALP (U/L, Range)	234 (49-1546)
Mean LDH (U/L, Range)	256 (77-1122)
RVI by tumor lesion (N, %)	
Involvement	32 (6.6)
Uninvolvement	450 (93.4)
Histological type (N, %)	
Osteoblastic	351 (72.8)
Chondroblastic	45 (9.3)
Fibroblastic	21 (4.4)
Other & non-classified	65 (13.5)
Type of operation (N, %)	
Limb-salvaging surgery	388 (80.5)
Amputation	94 (19.5)

ALP: Alkaline Phosphatase; LDH: Lactic Dehydrogenase; RVI: radiological vascular involvement.

**Table 2:** Univariate analysis of probable risk factors for 5-years OS and EFS

Variable	Patient (N)	5-year OS (%)	P-value	Std. (survival)	Patient (N)	5-year EFS (%)	P-value	Std. (survival)
**Gender**								
Male	298	66.2	0.383	5.68	298	48.1	0.598	5.78
Female	184	71.7		7.06	184	50.6		7.77
**Age (years)**								
≤14	160	61.2	0.094	4.54	160	44.4	0.288	8.32
>14	322	71.8		8.11	322	50.2		4.90
**Maximum diameter of tumor (mm)**								
≤80	278	79.9	<0.01	5.34	278	54.1	<0.01	6.19
>80	204	52.8		6.94	204	40.1		6.68
**ALP level**								
Normal	186	76.3	0.040	6.51	186	56.6	0.042	6.46
Elevated	296	63.3		5.95	296	43.3		6.02
**LDH level**								
Normal	310	72.9	0.060	5.50	310	50.7	0.146	5.98
Elevated	172	60.1		7.80	172	43.8		7.90
**Surgery**								
Amputation	94	56.5	0.112	10.29	94	37.5	0.052	10.05
Limb-salvage	388	71.0		5.00	388	50.7		5.30
**RVI by tumor lesion**								
Yes	32	44.8	<0.01	12.26	32	30.2	<0.01	11.85
No	450	71.7		4.77	450	50.9		5.13

ALP: Alkaline Phosphatase; LDH: Lactic Dehydrogenase; RVI: radiological vascular; OS: Overall survival; EFS: Event-free survival; Std.: standard deviation.

**Table 3:** Multivariate analysis of clinical factors for 5-year OS and EFS

Variable	Patient		5-year OS			5-year EFS		
	(N)		β-value	HR	95% CI	β-value	HR	95% CI
ALP level	Normal	186	0.452	1.57	0.96-2.56	0.374	1.45	1.00-2.11
	Elevated	296						
Maximum diameter of tumor	≤80	278	1.037	2.82	1.79-4.44	0.590	1.80	1.27-2.56
	>80	204						
RVI by tumor lesion	Yes	32	0.842	2.32	1.39-3.87	0.594	1.81	1.16-2.83
	No	450						

ALP: Alkaline Phosphatase; RVI: radiological vascular; OS: Overall survival; EFS: Event-free survival. Subtyping of Enneking IIB osteosarcoma based on the radiological relationship between the tumor and peritumoral reactive area with the main blood vessel.

**Table 4:** Baseline demographic characteristics in three radiological types

Characteristics	Type I	Type II	Type III	P-value
Mean age (Years, Range)	23 (11-47)	19 (7-47)	16 (8-29)	<0.05
**Gender (N, %)**				
Male	32 (69.6)	246 (60.9)	20 (62.5)	0.721
Female	14 (30.4)	158 (39.1)	12 (37.5)	
**Site of tumor (N, %)**				
Distal Femur	30 (65.2)	275 (68.1)	9 (28.1)	<0.05
Proximal Tibia	8 (17.4)	103 (25.5)	6 (18.8)	
Proximal Humerus	2 (4.3)	21 (5.2)	5 (15.6)	
Others	6 (13.1)	5 (1.2)	12 (37.5)	
**Mean ALP (U/L, Range)**	179 (52-397)	222 (49- 1546)	373 (77- 1011)	<0.05
Mean LDH (U/L, Range)	225 (86-681)	245 (77-1122)	336 (83-996)	<0.05
**Histological type (N, %)**				
Osteoblastic	33 (71.1)	279 (69.1)	23 (71.8)	0.398
Chondroblastic	4 (9.3)	35 (8.7)	3 (9.4)	
Fibroblastic	2 (4.4)	18 (4.5)	2 (6.3)	
Other & non-classified	7 (15.2)	72 (17.7)	4 (12.5)	
Mean maximum diameter of tumor (mm, Range)	63.2 (39-104)	79.1 (38-105)	84.0 (44-97)	<0.05
Mean NBR (cm, Range)	0.38 (0.31-0.58)	0.00	0.00	<0.05
Mean NBT (cm, Range)	1.07 (0.69-1.46)	0.35 (0.28-0.44)	0.00	<0.05
**Type of operation (N, %)**				
Limb-salvaging surgery	44 (95.7)	340 (84.2)	4 (12.5)	<0.05
Amputation	2 (4.3)	64 (15.8)	28 (87.5)	

ALP: Alkaline Phosphatase, LDH: Lactic Dehydrogenase, NBR: Nearest of blood vessels to reactive zone; NBT: Nearest of blood vessels to the tumor.

**Table 5:** Intraobserver and interobserver agreement of radiological classification

Type	Radiologist 1	Radiologist 2	Kappa
	First assessment		0.603
Type I	50	41	
Type II	400	406	
Type III	32	35	
	Second assessment		
Type I	57	39	0.586
Type II	390	400	
Type III	35	43	
Kappa	0.683	0.692	

## Abbreviations

CT: Computed tomography
PET-CT: Positron Emission Tomography-Computed Tomography
MRI: Magnetic resonance imaging
OS: Overall survival
EFS: Event-free survival
HR: Hazard ratio
ALP: Alkaline phosphatase
LDH: Lactic dehydrogenase
ANOVA: Analysis of variance
NCCN: National Comprehensive Cancer Network
AJCC: According to the American Joint Committee on Cancer
RVI: Radiological vascular involvement
NBR: Nearest of blood vessels to reactive zone
NBT: Nearest of blood vessels to the tumor
